# DNA repair mechanisms and human cytomegalovirus (HCMV) infection

**DOI:** 10.1007/s12223-014-0359-6

**Published:** 2014-11-01

**Authors:** Beata Smolarz, Jan Wilczyński, Dorota Nowakowska

**Affiliations:** Department of Fetal-Maternal Medicine and Gynaecology, Polish Mother’s Memorial Hospital Research Institute, 281/289 Rzgowska Street, Lodz, 93-338 Poland

## Abstract

Herpesvirus infections, such as those induced by human cytomegalovirus (HCMV), induce specific DNA damages. DNA damages can lead to cell mutation, death, apoptosis and immune system activation. Various types of DNA damage are repaired through multiple repair pathways, such as base excision, nucleotide excision, homologous recombination and nonhomologous end joining. Changes in the activity of DNA repair proteins during viral infection can cause disturbances in the DNA repair system and change its mechanisms. This report reviews results from studies, assaying a DNA repair system in HCMV infection.

## Introduction

DNA damage normally triggers activities of the cellular DNA repair machinery. There are six known systems of DNA repair: pathway of direct reversion of damage, base excision repair (BER), nucleotide-excision repair (NER), mismatch repair (MMR), homologous recombination (HR) and nonhomologous DNA end joining (NHEJ). Unrepaired DNA damage can lead to mutation, development of various diseases or cell death (Bartek et al. 2004; Jackson and Bartek 2009; Hoeijmakers 2009; Polo and Jackson 2011).

The viruses that induce cellular DNA damage include members of the herpesvirus group, adenovirus, mumps-virus, measles-virus, rubella-virus, poliovirus and papilloma-virus (Fortunato et al. 2000).

Human cytomegalovirus (HCMV) is a member of the betaherpesvirus subfamily and encodes more than 200 viral proteins (Chee et al. 1990; Salsman et al. 2008; Stern-Ginossar et al. 2012).

The viral infection process is very complex and driven by many mechanisms. It is known that HCMV can act as mutagen inducing various DNA damage (AbuBakar et al. 1988; Shen et al. 1997).

Herpesvirus infection is associated with apoptosis processes, immune activation, cell cycle control and DNA damage response (Chaurushiya and Weitzman 2009; Miller-Kittler and Sparer 2009; Weitzman et al. 2010).

It appears from literature that human cytomegalovirus enhances DNA repair capacity in host cells, without producing detectable lesions in cellular DNA or inhibiting DNA synthesis (Nishiyama and Rapp 1981).

Many studies have analysed cell repair capabilities, following viral infection. Those experimental studies focused on repairs of exogenously introduced damage in cellular DNA in the context of single viral protein expression (Becker et al. 1998; Prost et al. 1998; Groisman et al. 1999; Jia et al. 1999; Chipitsyna et al. 2004; Mathonnet et al. 2004; Liang et al. 2006; Sun et al. 2006; Trojanek et al. 2006; Durkin et al. 2008; Gruhne et al. 2009; Baydoun et al. 2011) and on effects of complete infection (Deng et al. 1992a; Philpott and Buehring 1999; Ranneberg-Nilsen et al. 2006; Duong et al. 2010; reviewed in Lilley et al. 2010; Pal et al. 2010; Kulkarni and Fortunato 2011).

Only several of those studies report some evidence of increased cell repair capacity after infection or viral protein overexpression (Chipitsyna et al. 2004; Kulkarni and Fortunato 2011; Ranneberg-Nilsen et al. 2006; Baydoun et al. 2011).

The control aspects of the five DNA repair mechanisms in virus-infected cells have not been well characterized. In this review, considerations and results of the experiments are presented, supporting the thesis on the important role of DNA repair systems in HCMV- infected cell.

## HCMV-related changes in base excision repair

The base excision repair (BER) pathway corrects most endogenous base lesions, including alkylation, oxidation and deamination, apurinic/apyrimidinic (AP) sites as well as single-strand breaks.

**Fig. 1:**
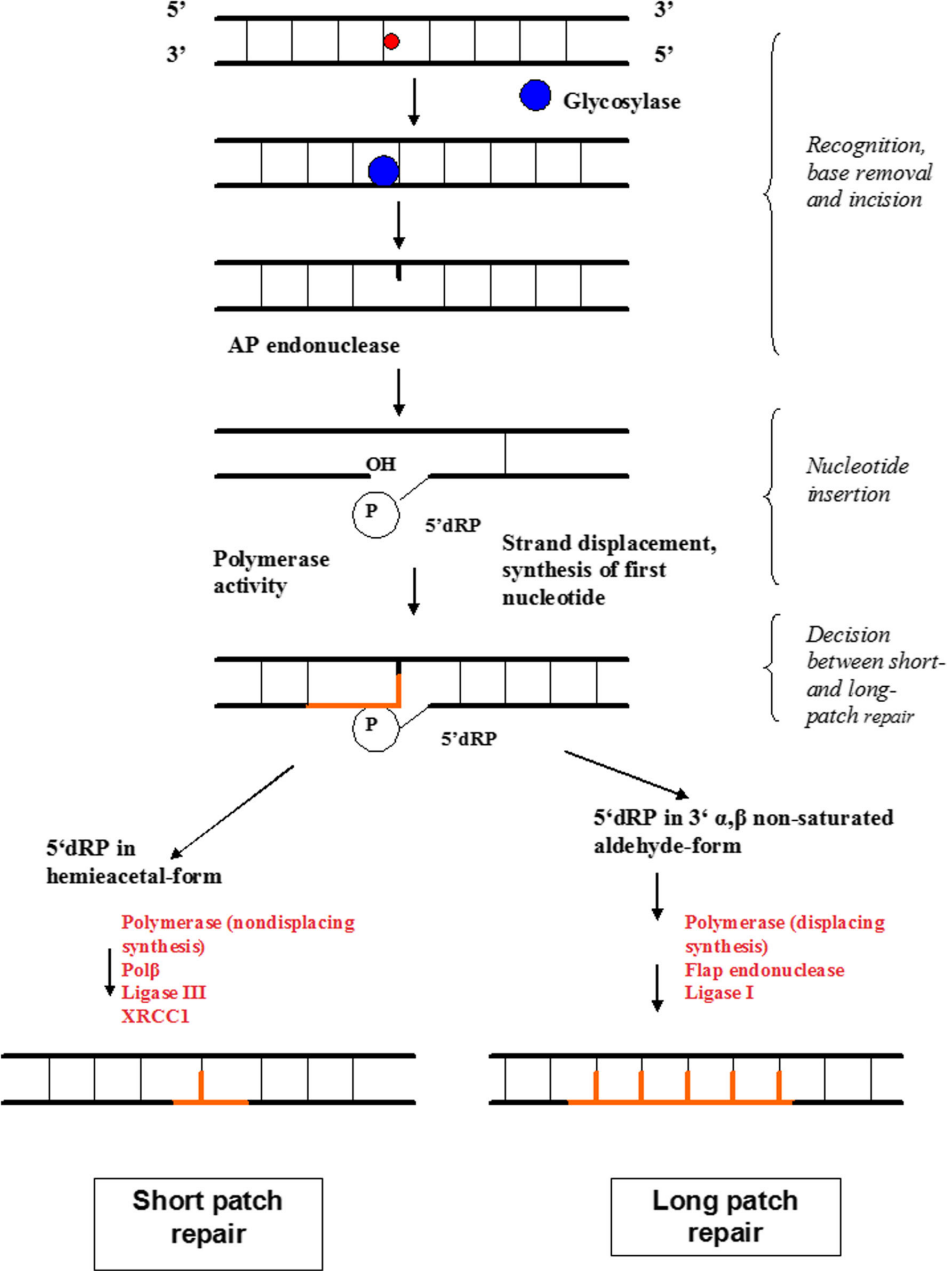
The base excision repair (BER) system. BER is initiated by a specific DNA glycosylases, which recognize and remove damaged or incorrect bases, creating AP sites. The AP endonucleases cleave an AP site to yield a 3′ hydroxyl adjacent to a 5′ deoxyribosephosphate (dRP). During short-patch BER, 5′dRP is displaced by DNA polymerase β (Polβ), which inserts a single nucleotide. Polβ is also involved in long-patch BER inserting the first nucleotide at reduced AP sites. The ligation step is performed by DNA ligases I and III. Ligase I interacts with Polβ and participates mainly in long-patch BER. DNA ligase III interacts with X-ray repair cross-complementing protein 1 (XRCC1) and Polβ and is involved only in short-patch BER

A damaged base is recognized by a specific DNA glycosylase, which cleaves the bond between the base and sugar, creating an abasic site (AP), which is a mutagenic and cytotoxic intermediate. The resulting AP site is further processed by the AP lyase activity associated with bifunctional DNA glycosylases and AP endonucleases cleaving 3′or 5′at the AP site, respectively. The processed AP sites are further repaired by two sub pathways of BER: short-patch BER, a mechanism, where only 1 nucleotide is replaced, or long-patch BER, where 2–13 nucleotides are replaced (Fig. 1) (Wilson and Bohr 2007; Almeida and Sobol 2007).

If not repaired by AP endonucleases in dividing cells, AP site may have dramatic consequences, leading to single and, subsequently, double DNA strand breaks, which are lethal to the cell (Evans et al. 2000).

It is known that HCMV infection is genotoxic to host cells (AbuBakar et al. 1988; Fortunato and Spector 2003; Nystad et al. 2008). Viral infection to cell is associated with production of reactive oxygen species (ROS).

Speir et al. (1998) reported that reactive oxygen metabolites were created soon after uptake of HCMV-infected smooth muscle cells, indicating a potentially genotoxic effect of viral infection.

Literature data demonstrate that modulation of BER activities may play some role in HCMV infection. Ranneberg-Nilsen et al. (2006) analysed the capability of HCMV-infected human embryonic lung fibroblasts to carry out base excision repair system. These researchers have demonstrated that some of the initial steps in the base excision repair machinery are downregulated during human cytomegalovirus replication in human fibroblasts.

Ranneberg-Nilsen et al. (2006) have investigated viral infection effects on the initial steps of BER, which are possessed by DNA glycosylases, cleaving the N-glycosylic bond and subsequent incision of the phosphodiester bond at the abasic site by an AP endonuclease activity. It was shown that the DNA glycosylase activity for removal of oxidized and alkylated bases declined in HCMV-infected cells.

Moreover, the AP endonuclease activity was enhanced in HCMV-infected cells. However, the expression level of the major human AP endonuclease (APE1) was not altered, indicating that either HCMV had its own AP endonuclease activity or that APE1 could have been subjected to post-translation modifications that could modulate its activity.

Furthermore, they speculate that reduced BER system activity in HCMV-infected cells may contribute to increased genomic variation of the virus.

An experimental analysis has provided evidence that increased excision rates of uracil and enhanced AP endonuclease activity in infected cells may form gaps in viral DNA, required for efficient viral DNA synthesis at late stages of viral replication (Ranneberg-Nilsen et al. 2006).

In conclusion, Ranneberg-Nilsen et al. (2006) demonstrated an upregulation of base excision repair system activities that could be involved in HCMV replication, whereas other BER activities, such as glycosylase activities, initiating repairs of alkylated and oxidized bases, were downregulated in viral-infected cells.

Future studies will be aimed at clarifying the functions of BER during HCMV infection.

## HCMV-related changes in nucleotide excision repair

**Fig. 2:**
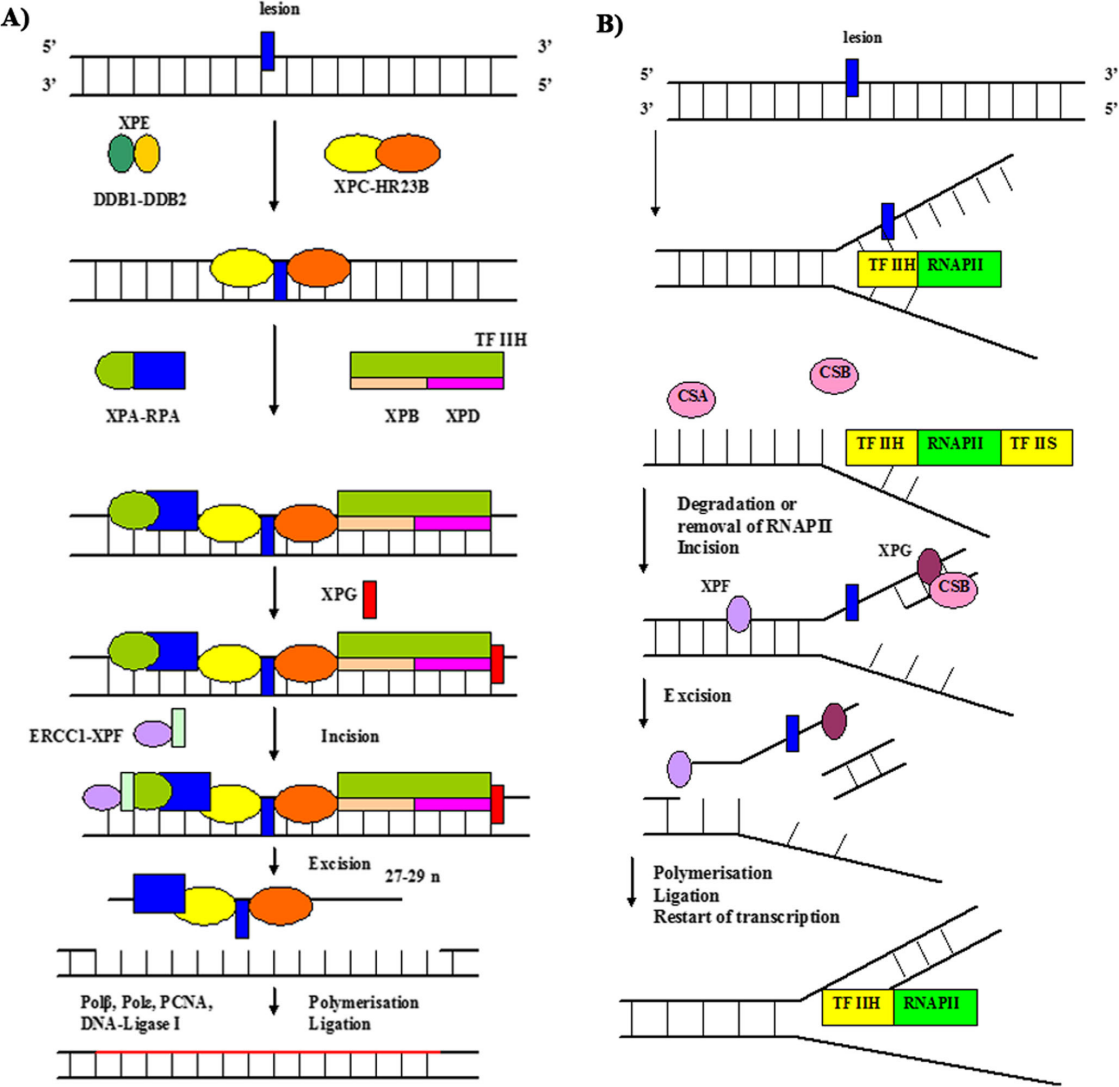
The nucleotide excision repair. **a** Global genomic repair (GGR), xeroderma pigmentosum, complementation group C (*XPC*), UV excision repair protein RAD23 homolog B (*HR23B*), replication protein A (*RPA*), xeroderma pigmentosum group A (*XPA*) or DNA damage-binding protein 1 and 2 (*DDB1*–*DDB2*) complex recognition DNA damage. DNA repair is performed by the transcription factor TFIIH, and excision of the lesion by xeroderma pigmentosum, complementation group G and F (*XPG* and *XPF*), excision repair cross-complementing 1 (*ERCC1*). Finally, resynthesis occurs by polymerase (*Polδ* or *Polϵ*) and ligation by DNA ligase I. **b** Transcription-coupled repair (*TCR*). First step, RNA polII (*RNAPII*) is blocked. This leads to assembly of Cockayne syndrome group A/B proteins (*CSA*, *CSB*) and/or the transcription elongation factor TFIIS at the site of the lesion, by which RNAPII is removed from the DNA or displaced from the lesion, making it accessible to the exonucleases XPF. Second step, XPG cleaving the lesion-containing DNA strand. Third step, resynthesis again occurs by Polδ or Polϵ and ligation by DNA ligase I

The nucleotide excision repair system removes short DNA, damaged base-containing oligonucleotides (Hanawalt 2002). NER recognizes bulky lesions, caused by carcinogenic compounds, and covalent linkages between adjacent pyrimidines, resulting from exposure to UV. NER is a multistep process, involving numerous proteins, and is classified into global genome repair (GG–NER) that occurs in the genome, and transcription-coupled repair (TCR), which removes lesions in the transcribed strand of active genes (Fig. 2).

Because NER is involved in removing a substantial number of DNA damages, which can contribute to the genome instability, it is reasonable to check whether variability in repair efficiency may be associated with HCMV infection.

What is important, many reports introduce the role of NER systems in the development of viral infection. Those analyses targeted the expression of single viral proteins in evaluation of UV-induced damage. The results suggest that the expression of the hepatitis B X protein (HBX) in different cell types (Becker et al. 1998; Groisman et al. 1999; Jia et al. 1999; Mathonnet et al. 2004) or the expression of Epstein Barr virus proteins EBNA3C or LMP1 in transfected cells (Gruhne et al. 2009), decreased repair efficiency of UV-induced damage in transfected cells.

In the literature, recent studies are described, examining interactions with the NER machinery and/or with HCMV infection’s influence on cellular damage repair. These reports demonstrate that NER may play an important role during HCMV entry to cells (O’Dowd et al. 2012). It appears from the reports that differential association of certain cellular repair proteins, e.g. XP proteins with HCMV, may have far-reaching implications in the pathogenesis of viral infection (O’Dowd et al. 2012).

An in vitro analysis of infected fibroblasts revealed selective removal of UV lesions from the viral, and not cellular, DNA within infected cells (O’Dowd et al. 2012).

O’Dowd et al. exposed infected fibroblasts to ultraviolet (UV) radiation to inflict DNA damage. The ultraviolet radiation phenomenon is known to fuse adjacent thymine bases together into dimers, which are then cut out and replaced through NER system. Experimental analysis by comet assays technique showed that repair was initiated but not completed in the infected cells. The cyclobutane pyrimidine dimers (CPDs) were localized by immunofluorescence. CPDs were significantly reduced in viral DNA but were unchanged in the infected host DNA after a day of repair. These CPDs were efficiently repaired from viral DNA but not from the host cellular DNA suggesting that host genomes are unable to repair DNA damage (O’Dowd et al. 2012).

Recent studies demonstrate that a nucleotide excision repair-associated factor, DNA damage binding protein 2 (DDB2), is required for efficient HCMV DNA replication (Xiaofei et al. 2014). DDB2 is a component of a Cul4A-Ub ligase complex that participates in nucleotide excision repair. The DDB1-CUL4ADDB2 complex is a cullin-RING (i.e. E3) Ub-ligase that targets histone H2A at UV-damaged DNA sites (Xiaofei and Kowalik 2014).

In conclusion, little is provided in the literature about the role of nucleotide excision repair mechanisms during human cytomegalovirus infection. Future HCMV research should reveal more interesting, NER-modulating viral effectors.

## HCMV-related changes in mismatch repair

**Fig. 3:**
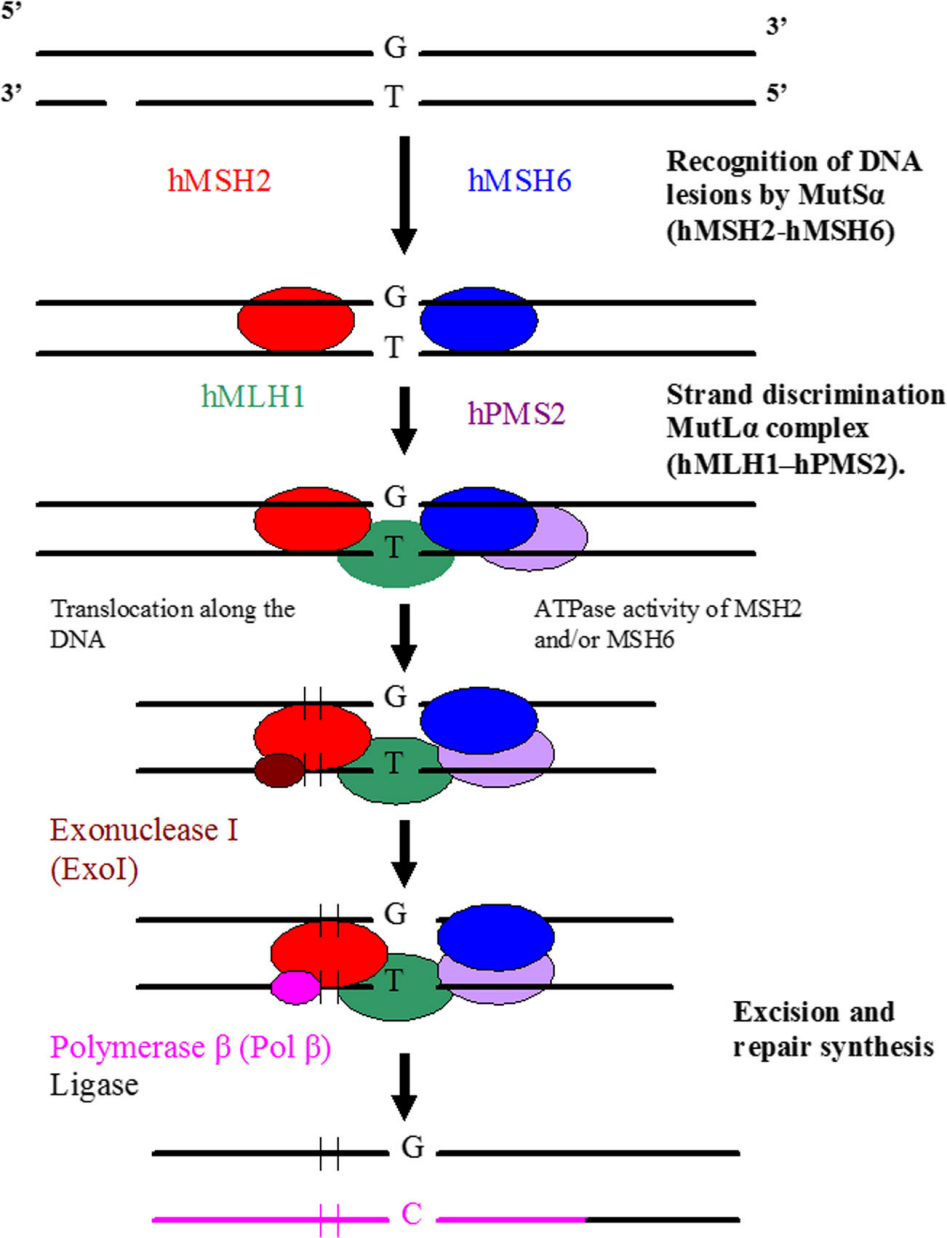
The mismatch repair (MMR). Recognition of DNA lesions occurs by heterodimer hMSH2–hMSH6 (MutSα). Upon binding to the mismatch, MutSα associates with heterodimeric complex hMLH1-hPMS2 (MutLα). After formation of a complex composed of MutSα and MutLα, excision is performed by ExoI and repair synthesis by Polβ

Mismatch repair removes mispaired bases, resulting from replication errors, recombination between imperfectly matched sequences and deamination of 5-methyl-cytosine. Mismatch repair have escaped the proofreading function of the DNA polymerase and insertion/deletion (IDLs). The main MMR pathway is initiated by mismatch recognition by the heterodimer, consisting of MSH2 and MSH6 proteins (also called MutSα) (Fig. 3). MutSα is responsible for the recognition of base mismatches and insertion/deletion (IDLs) in mono to tetranucleotide repeats. This complex, i.e. MutSα, is able to recognize the majority of base–base mismatches and short IDLs (Hsieh and Yamane 2008). MMR is a highly conserved repair pathway that functions in improving replication fidelity by correcting replication-associated base–base and insertion/deletion mispairs. The MMR mechanism plays an important role in repair of oxidative damage by some mechanisms that are not well understood (Skinner and Turker 2005). MMR is essential for the maintenance of genome stability (Karran 1996; Ben Yehuda et al. 2000; Neri et al. 2005; Krichevsky et al. 2004).

To date, no studies have addressed the association between alterations in the MMR system during human cytomegalovirus infection. Changes in the MMR system during HCMV infection are not evident and further investigations are necessary, concerning its efficiency in infected organisms.

## HCMV-related changes in double-strand break repair

Out of all the DNA damages, double-strand breaks (DSBs) are most mortal to cell. If not repaired, they cause losses of chromosomes and cell death. DSB accumulation destabilizes the genome and rearranges it, leading to transcription downregulation and development of various diseases (Jackson 2002).

HCMV can inflict site-specific chromosomal damage in a form of DSBs. Disruption of DSB repair system often leads to more intensive viral infection (Fortunato et al. 2000; Fortunato and Spector 2003).

**Fig. 4:**
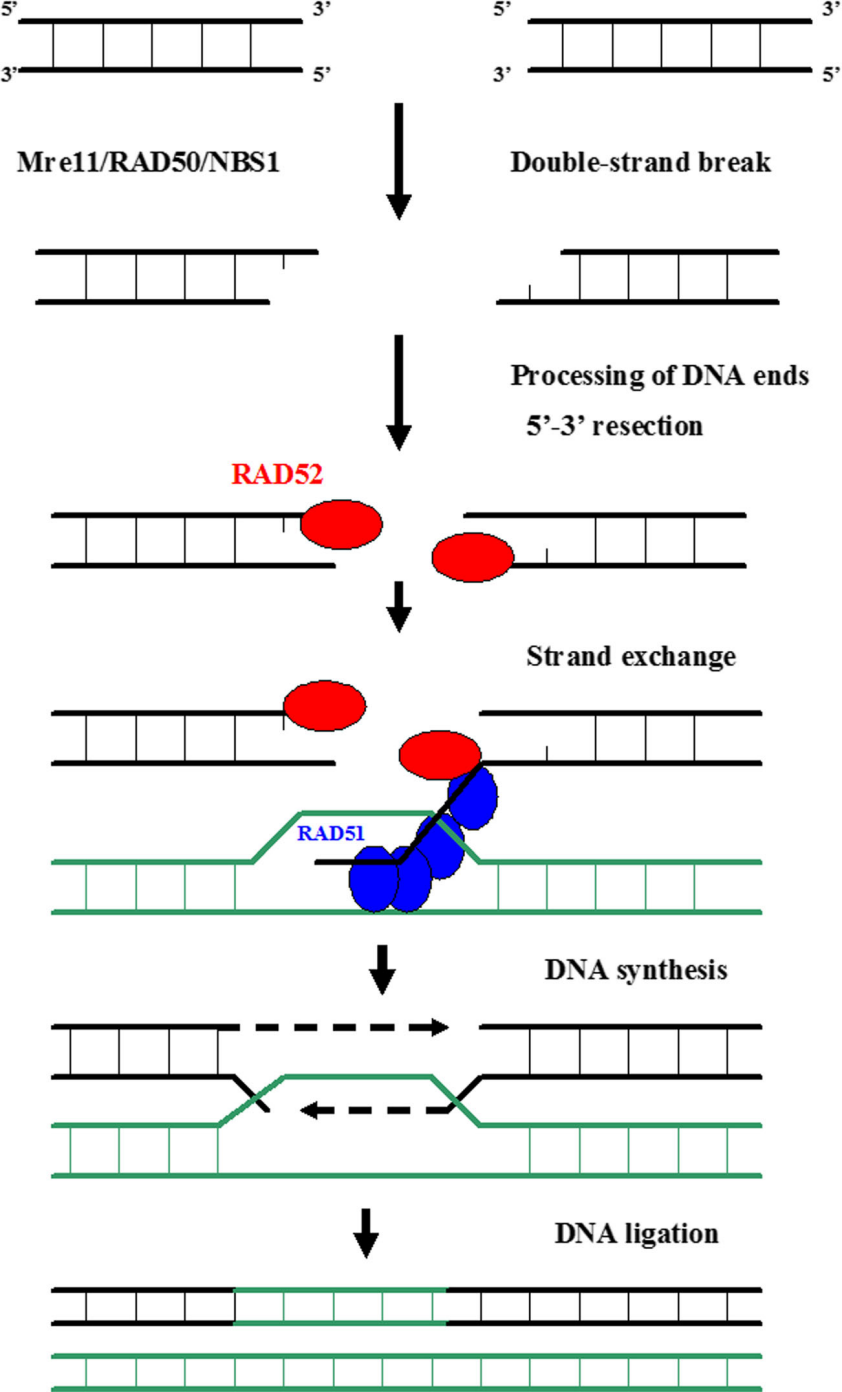
The homologous recombination (HR) system. HR starts with nucleolytic resection of the double strand break in the 5′ → 3′ direction by the MRE11–Rad50–NBS1 (MRN) complex, forming a 3′ single-stranded DNA fragment to which RAD52 homolog (*S. cerevisiae*) (Rad52) binds. Rad52 interacts with Rad51 homolog (RecA homolog, *E. coli*) (*S. cerevisiae*) (Rad51), provoking a DNA strand exchange with the undamaged, homologous DNA molecule. After DNA synthesis, ligation and branch migration, the resulting structure is resolved

**Fig. 5:**
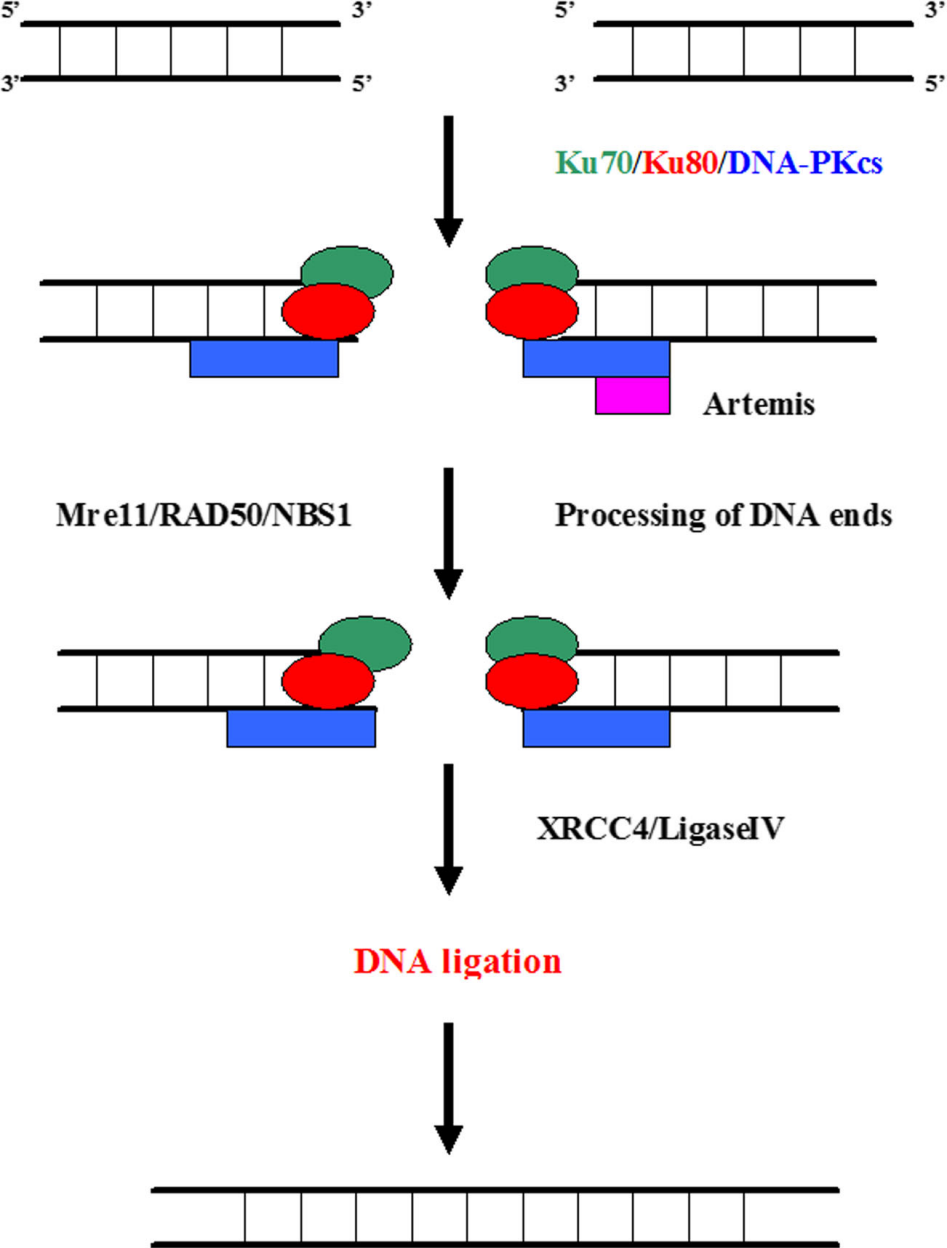
The non-homologous end joining (NHEJ). The first step in NHEJ is the binding of a heterodimeric complex consisting of the proteins Ku70 and Ku80. Ku heterodimer associates with the catalytic subunit of DNA-dependent protein kinase (DNA–PKcs), forming the DNA–PK holoenzyme. One of the targets of DNA–PKcs is X-ray repair cross-complementing protein 4 (XRCC4). XRCC4 forms a stable complex with DNA ligase IV. DNA–PK activates XRCC4–ligase IV, which links the broken DNA ends together. Before re-ligation by XRCC4–ligase IV, the DNA ends are processed by the MRE11–Rad50–NBS1 complex, presumably involving Artemis

The DSB repair system functions via two mechanisms: homologous recombination (HR) and nonhomologous DNA end joining (NHEJ) (Figs. 4, and 5) (Jackson 2002; Helleday 2003).

The nonhomologous end-joining pathway simply fuses two broken ends with little or no regard for sequence homology. It is known that NHEJ is involved in the immunological system maturation, cell–cell and cell–matrix adhesion (Tonegawa 1983).

The activities of NHEJ factors, such as Ku70/80, Artemis and Cernunnos protein, are very important for the immune system development (Weterings and van Gent 2004; Moshous et al. 2001; Buck et al. 2006)

Defects of NHEJ, observed during B and T lymphocyte receptors maturation, may be associated with immunodeficiency. The defective immune system is correlated with easy viral infection and HCMV diseases (Gamadia et al. 2003).

It appears from literature that a single viral protein can modulate homologous recombination repair (Nakai-Murakami et al. 2007; Trojanek et al. 2006; Kulkarni and Fortunato 2011).

Rad51 homolog (RecA homolog, *Escherichia coli*) (*Saccharomyces cerevisiae*) is involved in the homologous recombination and repair of double-strand breaks in DNA and DNA cross-links and in the maintenance of chromosome stability.

Experimental studies revealed Rad51 levels to be significantly increased in HCMV-infected human foreskin fibroblasts (HFFs) (Luo et al. 2007).

Literature data suggest that Rad51 levels do not generally increase in normal cells (Haaf et al. 1995). Raderschall et al. (2002) showed that increased RAD51 levels in tumour cells were found to be associated with unplanned HR and genetic instability. Therefore, elevated RAD51 levels may be signalling extensive DNA damage.

Human cytomegalovirus infection and expression may be specifically associated with human cancers, including malignant glioma, colorectal and prostate cancer (Cobbs et al. 2002; Harkins et al. 2002; Samanta et al. 2003, reviewed in Michaelis et al. 2009; Dziurzynski et al. 2012). HCMV can deregulate the signalling pathways, involved in initiation and promotion of various malignancy, including tumour suppressor, mitogenic signalling, inflammatory, immune regulation, angiogenesis and invasion. Human cytomegalovirus might play an important role in modulating tumour microenvironment as well as in the initiation and promotion of tumour cell development (Soroceanu and Cobbs 2011). The significance of HCMV infection in oncogenesis is an active area of scientific research (Cinatl et al. 2004). Although still controversial, there is a growing body of evidence that links viral infection to a various malignancy.

It is supposed that HCMV propensity to cause cancer may be associated with deregulation of homologous recombination of DNA repair machinery.

During HCMV infection, DSB repairing functions occur, leading to increased cancer development in infected cells because of genome rearrangement (Dziurzynski et al. 2011; Michaelis et al. 2011).

The oncogenic potential of human adenoviruses and HCMV are associated with site-specific chromosomal damage. HCMV induces specific damage in chromosome 1 at two loci 1q23 and 1q42 (Fortunato et al. 2000; Nystad et al. 2008).

These HCMV effects on chromosomal integrity and the synergistic activity of HCMV infection in combination with other cytotoxic agents contribute significantly to global genetic instability, which is a major cancer promoter (Albrecht et al. 2004; Siew et al. 2009). It has been shown that the HCMV protein UL76 can induce chromosomal aberrations (e.g. production of micronuclei, misaligned chromosomes, chromosomal lagging and bridging) (Siew et al. 2009).

Cellular responses to DNA damage are mediated by a number of protein kinases, including ATM (ataxia telangiectasia-mutated) and ATR (ATM and Rad3-related). HCMV has been shown to disrupt DNA repair pathways, including the activity of ATM and ATR (Luo et al. 2007).

HCMV interacts with your host DNA damage response (DDR) signalling molecules and repair machinery. Interactions between HCMV-encoded proteins pUL35 and pUL27 and DNA-damage DNA-binding protein (DDB1) contribute to DDR activation (Salsman et al. 2012; Reitsma et al. 2011; Costa et al. 2013; reviewed in Xiaofei and Kowalik 2014).

pUL35 can active DDR, causing γH2AX and 53BP1 foci formation and induce a cell cycle arrest which likely supports viral replication (Salsman et al. 2012). HCMV, in particular, encodes IE1, IE2, pp71, pUL97, pUL69 proteins that both modulate cell cycle controls and the host DDR to promote viral replication. The inactivation of RB family members by these four proteins and subsequent deregulation of E2F1 appears to result in double-strand breaks in human fibroblasts (Xiaofie et al. 2011). The mechanism by which E2F1 stimulates host DDR is not well understood.

It is known that viruses utilize initial DDR response to optimize infection (Weitzman et al. 2010; Weller 2010). HCMV genome replication requires host DDRs and raises the possibility that DNA repair pathways may influence viral replication. A recent study shows that a DNA repair factor, DDB2, can contribute to HCMV replication (Xiaofei et al. 2014).

Literature data have shown that in HCMV-infected human foreskin fibroblasts, DDR is activated at the time of viral deposition and during late-phase replication (Gaspar and Shenk 2006; Luo et al. 2007).

The progression of the HCMV life cycle also depends on the capability of the virus to modulate host cell processes such as cellular gene expression and apoptosis process to its advantage. Recently, US27 was identified as a modulator of cellular gene expression (Arnolds et al. 2013). The UL28-UL29, UL38, UL79 and RNA2.7 were found to control apoptosis (Terhune et al. 2007; Moorman et al. 2008; Siew et al. 2009; Qian et al. 2011; Fliss and Brune 2012; Costa et al. 2013; Savaryn et al. 2013).

In conclusion, several experimental studies have shown that double-strand break (DSB) repair undergoes viral-related changes, which are likely to contribute to the accumulation of DNA lesions.

## DNA repair gene polymorphism and HCMV infection

As mentioned above, the human cytomegalovirus can inflict site-specific chromosomal damage in form of double-strand breaks (DSBs). The cellular reaction to the DNA damaging agents can modulate susceptibility to viral infection. This reaction is mainly determined by the efficacy of DNA repair which, in turn, may be influenced by the variability of DNA repair genes, expressed by their polymorphism.

DNA repair genes are highly polymorphic in nature. However, little is known about HCMV infection-related DNA repair gene polymorphisms.

Human cytomegalovirus infection and expression may be specifically associated with human cancers, including colon carcinoma, prostate, adenocarcinoma, cervical carcinoma, neuroblastoma and glioblastoma (Harkins et al. 2002; Samanta et al. 2003; Cobbs et al. 2002; Cinatl et al. 2004; reviewed in Michaelis et al. 2009; Dziurzynski et al. 2012).

Polymorphisms in DNA repair genes may alter the activity of the proteins and, thus, modulate susceptibility to cancer (Karahalil et al. 2012; Samulak et al. 2011; Smolarz et al. 2013).

It is supposed that HCMV propensity to cause cancer may be associated with DNA repair genes, previously implicated in tumour disease. Therefore, an effective analysis of DNA repair gene polymorphism during viral infection may represent a true advance in the study of viral pathogenesis.
